# The Obesity Paradox in Major Adverse Cardiovascular Events After PCI for Acute Coronary Syndrome: A Narrative Review

**DOI:** 10.3390/jcdd13060251

**Published:** 2026-06-05

**Authors:** Lisa Simioni, Wesley Bennar, Giulia S. Beretta, Thais Pittet, Giacomo Maria Cioffi, Julius Jelisejevas, Peter Wenaweser, Pascal Meier, Serban Puricel, Mario Togni, Stéphane Cook, Ioannis Skalidis

**Affiliations:** Department of Cardiology, HFR—Fribourg Cantonal Hospital and University, 1708 Fribourg, Switzerland; lisa.simioni@h-fr.ch (L.S.); wesley.bennar@h-fr.ch (W.B.); giulia.beretta@h-fr.ch (G.S.B.); thais.pittet@h-fr.ch (T.P.); giacomomaria.cioffi@h-fr.ch (G.M.C.); juliusjonas.jelisejevas@h-fr.ch (J.J.); peter.wenaweser@h-fr.ch (P.W.); pascal.meier@h-fr.ch (P.M.); serban-george.puricel@h-fr.ch (S.P.); mario.togni@unifr.ch (M.T.); stephane.cook@unifr.ch (S.C.)

**Keywords:** obesity paradox, obesity, major adverse cardiovascular events, percutaneous coronary intervention, acute coronary syndrome, STEMI

## Abstract

Background: Obesity is increasing worldwide and remains a major contributor to cardiovascular morbidity and mortality. It is strongly associated with hypertension, dyslipidemia, diabetes mellitus, endothelial dysfunction, and chronic inflammation, all of which promote coronary artery disease and acute coronary syndrome (ACS). Despite this well-established risk profile, multiple studies have described an “obesity paradox,” suggesting that obese patients may experience better outcomes after percutaneous coronary intervention (PCI) for ACS than normal-weight individuals. Objective: This narrative review aims to discuss the pathophysiological basis of the obesity paradox and to synthesize contemporary evidence regarding the relationship between body mass index (BMI), major adverse cardiovascular events (MACE), and mortality after PCI in patients presenting with ACS. Results: Contemporary observational cohorts consistently suggest a non-linear relationship between BMI and MACE outcomes after PCI. Overweight and mildly obese patients often demonstrate lower crude mortality and fewer MACE, whereas underweight patients consistently show the poorest prognosis. However, after adjustment for age, left ventricular ejection fraction (LVEF), renal function, frailty, and nutritional status, obesity is less consistently associated with improved outcomes. Overweight status appears to be more reproducibly associated with better prognosis than obesity itself. Conclusions: The obesity paradox is likely driven less by a true protective effect of excess adiposity and more by younger age at presentation, preserved physiological reserve, lower frailty burden, and the limitations of BMI as a marker of cardiovascular risk. Underweight status emerges as the strongest predictor of adverse outcomes. Nutritional assessment and body composition should complement BMI in risk stratification after ACS.

## 1. Introduction

Obesity is a growing public health burden worldwide. In 2022, 43% of the Swiss population was overweight (30.9%) or obese (12.1%), an increase of nearly 13% in 30 years [1]. Meanwhile, the prevalence of obesity more than doubled globally between 1990 and 2022 [2]. In Switzerland, this problem affects men more than women [1]. This metabolic condition, defined by a body mass index (BMI) ≥ 30 kg/m^2^ according to the recommendations of the World Health Organization (WHO), predisposes individuals to multiple cardiovascular diseases [2].

Obesity plays a central role in the development of coronary artery disease through a complex network of pathophysiological mechanisms, including insulin resistance, chronic low-grade inflammation, endothelial dysfunction, dyslipidemia, hypertension, and prothrombotic activation. These alterations accelerate atherosclerosis and significantly increase the risk of acute coronary syndrome (ACS), including ST-segment elevation myocardial infarction (STEMI), non-ST-segment elevation myocardial infarction (NSTEMI), and unstable angina. As a result, obesity is highly prevalent among patients undergoing percutaneous coronary intervention (PCI) for ACS [3,4].

Despite the well-established adverse cardiovascular effects of obesity, numerous studies have described a paradoxical association between increased BMI and improved clinical outcomes after PCI for ACS. This unexpected observation has given rise to the concept of the “obesity paradox”. First popularized by Carl J. Lavie and colleagues [5] in 2003, this concept suggests that overweight and obese patients undergoing PCI after ACS may experience lower mortality and fewer major adverse cardiovascular events (MACE) than normal-weight individuals. Interestingly, this protective association appears to be more consistently observed in overweight and mildly obese patients, whereas underweight patients often demonstrate the poorest prognosis [6,7].

Multiple hypotheses have been proposed to explain this paradox. Obese patients may benefit from greater metabolic and nutritional reserve during acute illness, better tolerance to catabolic stress, and protection against cachexia and sarcopenia [8]. Furthermore, these patients appears to have larger coronary arteries size, facilitating stent placement and reducing the risk of post-PCI complications [9].

They also tend to present with ACS at a younger age and often have better preserved left ventricular ejection function (LVEF) at the time of intervention [10,11]. In addition, adipose tissue functions as an active endocrine organ capable of secreting adipokines that may exert complex anti-inflammatory and cardioprotective effects during acute myocardial injury [12].

However, the validity of the obesity paradox remains highly controversial. BMI alone is an imperfect marker of adiposity levels and does not distinguish between visceral fat, lean body mass, or nutritional status [13,14]. Several studies suggest that the apparent survival advantage of obesity may be largely explained by residual confounding, reverse causality, survival bias, and the particularly poor prognosis of frail or underweight patients rather than by a true protective effect of excess adiposity itself. Central obesity and body composition may therefore be more relevant prognostic markers than BMI alone.

In patients undergoing PCI for ACS, understanding whether obesity independently influences post-procedural outcomes has important clinical implications for risk stratification, nutritional assessment, and long-term secondary prevention. Moreover, more recently, epicardial adipose tissue (EAT) has emerged as a potentially important mediator of cardiovascular risk [15,16]. Although physiological EAT may exert local cardioprotective effects, pathological expansion of this depot is associated with coronary inflammation, plaque vulnerability, and adverse cardiovascular outcomes [15].

This narrative review aims to examine the pathophysiological mechanisms underlying the obesity paradox and to discuss contemporary evidence regarding the association between obesity, BMI categories, and MACE after PCI in patients presenting with ACS.

## 2. Methodology

This manuscript was designed as a narrative review aiming to provide a clinically oriented overview of the obesity paradox in patients undergoing PCI after ACS. The objective was to synthesize the most relevant contemporary evidence and to discuss the pathophysiological mechanisms and clinical implications associated with BMI and MACE.

A targeted literature review was performed focusing on original studies involving adult patients with ACS treated with PCI and reporting cardiovascular outcomes according to BMI categories. Priority was given to original studies involving adult patients with ACS treated with PCI and reporting cardiovascular outcomes according to BMI categories. Particular attention was paid to studies evaluating mortality, MACE, and the prognostic impact of overweight, obesity, underweight status, nutritional status, and frailty.

Given the narrative nature of this review, no formal systematic search strategy, study quality scoring, or quantitative meta-analysis was performed. Instead, the selected studies were critically reviewed and synthesized to provide a comprehensive overview of the available evidence, highlight areas of consistency and controversy, and discuss the limitations of BMI as a measure of cardiovascular risk.

During manuscript preparation, ChatGPT (GPT-5.3; OpenAI, San Francisco, CA, USA) was used exclusively for English language editing and improvement of clarity and readability. All scientific content, critical interpretation, and final manuscript preparation were performed independently by the authors.

## 3. Pathophysiological Basis of the Obesity Paradox

The relationship between obesity and cardiovascular outcomes after ACS is complex and cannot be explained by BMI as a standalone metric. While obesity is a well-established risk factor for the development of coronary artery disease, multiple clinical studies have paradoxically reported improved outcomes in overweight and mildly obese patients after PCI. Understanding this apparent contradiction requires a broader pathophysiological perspective integrating both the harmful chronic effects of obesity and the potential influence of confounding factors that may contribute to the seemingly protective effect of obesity observed during ACS.

### 3.1. Obesity as a Cardiovascular Risk Factor

Obesity is well established as a risk factor for the development of cardiovascular diseases. This condition promotes the development of atherosclerotic cardiovascular disease through multiple interrelated mechanisms involving metabolic dysfunction, chronic inflammation, endothelial injury, and neurohormonal activation [3,4,17]. Excess adipose tissue, particularly visceral fat, acts as an active endocrine organ rather than a simple energy storage compartment. The adipose tissue secretes numerous adipokines and pro-inflammatory cytokines, including leptin, resistin, tumor necrosis factor-alpha, and interleukin-6, which contribute to systemic inflammation, oxidative stress, endothelial dysfunction, and vascular remodeling [12,18,19,20].

In parallel, obesity is characterized by a constellation of cardiometabolic abnormalities—including insulin resistance, dyslipidemia, hypertension, and type 2 diabetes mellitus—that collectively accelerate atherosclerotic disease progression and plaque destabilization [3,4,12,17]. Neurohormonal activation, particularly involving the sympathetic nervous system and the renin–angiotensin–aldosterone system, further exacerbates myocardial remodeling and ventricular dysfunction [3,17]. Moreover, obesity-associated conditions such as obstructive sleep apnea syndrome and physical inactivity further amplify these pathophysiological mechanisms. Obesity is also characterized by a prothrombotic and procoagulant state, with increased platelet activation, impaired fibrinolysis, and elevated circulating levels of inflammatory mediators, thereby increasing the risk of coronary thrombosis during ACS [4,17]. These phenomena can be explained by the increased production of bioactive substances such as leptin, adiponectin, TNF-α, IL-6, and resistin, which directly or indirectly affect platelet function [3]. Moreover, obese individuals exhibit increased secretion of PAI-1 (plasminogen activator inhibitor-1) and TAFI (thrombin-activatable fibrinolysis inhibitor), contributing to impaired fibrinolytic activity [21,22]. In addition, the platelet cytoskeleton in obese patients appears to undergo structural reorganization, with overexpression of certain peptides involved in the synthesis of surface proteins required for platelet adhesion and activation [23].

From a myocardial and cardiometabolic perspective, excessive fatty acid availability may further impair cardiac function. Free fatty acids constitute a less efficient energy substrate for the myocardium because their oxidation requires greater oxygen consumption to generate equivalent amounts of adenosine triphosphate (ATP), resulting in reduced mechanical efficiency [24]. Chronic fatty acid overload also promotes lipotoxicity [25]. Once the myocardium exceeds its capacity to store lipids safely, excess fatty acids exert deleterious effects on mitochondria, impairing cellular energy production [26].

EAT has emerged as a particularly relevant adipose depot for cardiovascular risk assessment and may provide additional insights into the mechanisms linking obesity to adverse cardiovascular outcomes beyond BMI-based classification [15]. Located between the myocardium and the visceral pericardium, EAT is in direct anatomical contact with the coronary arteries and myocardium without any fascial barrier [27]. Under physiological conditions, EAT exerts several cardioprotective functions, including mechanical cushioning, local energy supply through free fatty acid metabolism, thermogenic activity resembling brown adipose tissue, and secretion of anti-inflammatory adipokines [15,28]. However, in obesity and metabolic disease, EAT undergoes substantial remodeling characterized by increased volume and a shift toward a pro-inflammatory, pro-fibrotic, and insulin-resistant phenotype [16].

Pathological EAT secretes increased amounts of pro-inflammatory mediators, including TNF-α, IL-6, MCP-1, and resistin, thereby promoting oxidative stress, endothelial dysfunction, and vascular inflammation [16,28]. Through paracrine and vasocrine signaling, EAT may directly contribute to coronary plaque development and instability, with greater EAT thickness being associated with increased atherosclerotic burden [29].

Beyond its vascular effects, EAT contributes to myocardial remodeling and has been independently associated with MACE and adverse long-term outcomes following ACS and PCI [29]. These findings suggest that EAT may provide a more accurate reflection of cardiovascular risk than BMI alone, highlighting the importance of body fat distribution and adipose tissue quality in the interpretation of the obesity paradox. Furthermore, EAT has emerged as a promising therapeutic target for cardiometabolic interventions, including GLP-1 receptor agonists and SGLT2 inhibitors [28,30].

In addition, calcium homeostasis becomes disrupted through increased lysophosphatidylinositol metabolism, leading to excessive Ca^2+^ release from the sarcoplasmic reticulum and subsequent impairment of cardiac contractility [31]. Furthermore, as the heart is also a muscular organ, insulin resistance similarly affects cardiomyocytes and contributes to adverse left ventricular remodeling and mitochondrial dysfunction. These alterations involve changes ranging from the activation of proximal insulin signaling pathways to the repression of glucose transport, ultimately reducing the myocardium’s ability to efficiently utilize its preferred energy substrate [24,32,33].

**Figure 1:**
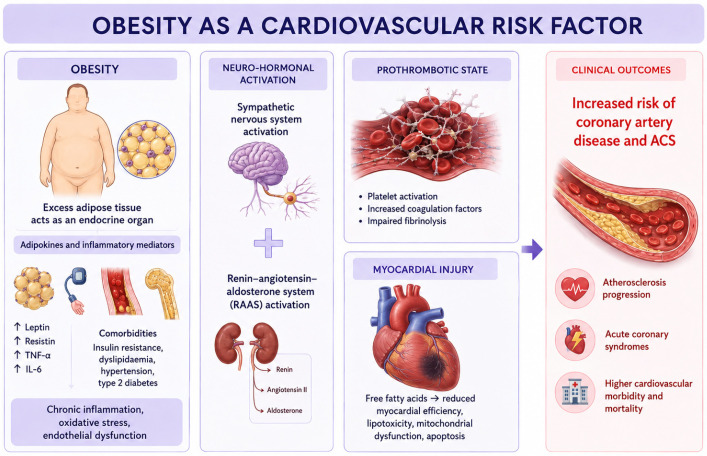
Pathophysiological mechanisms linking obesity to cardiovascular disease and acute coronary syndromes. Obesity leads to excess adipose tissue, particularly in visceral and epicardial location. This adipose tissue secretes bioactive mediators that accelerate atherogenesis, impair myocardial metabolism, and contribute to adverse cardiac remodeling. Thus, obesity promotes atherosclerotic cardiovascular disease through chronic inflammation, endothelial dysfunction, insulin resistance, neurohormonal activation, and a prothrombotic state. These mechanisms increase the risk of coronary artery disease and ACS. ACS = acute coronary syndrome, TNF-α = tumor necrosis factor alpha, IL-6 = Interleukin 6.

These mechanisms explain why obesity substantially increases the lifetime risk of coronary artery disease and ACS. However, they do not explain why patients with higher BMI may sometimes appear to have better outcomes after PCI, giving rise to the concept of the obesity paradox. To provide a clearer overview of these findings, a summary figure is presented below (see Figure 1).

### 3.2. Potential Mechanisms Supporting the Obesity Paradox

Several mechanisms may explain why overweight and mildly obese patients appear to experience better outcomes after ACS. First, obese patients have larger energy and protein reserves, which may protect against acute catabolic stress, cachexia, and malnutrition during hospitalization and recovery [8]. Moreover, during metabolic stress conditions such as prolonged fasting, obese individuals exhibit increased total lipolysis, reduced urea production, and lower muscle proteolysis compared with lean individuals. This metabolic profile suggests better preservation of muscle protein stores through the preferential mobilization of lipid reserves [34]. This greater metabolic reserve may improve tolerance to ischemic injury and invasive procedures.

Second, obese patients generally present with ACS at a younger age than leaner individuals [7,35]. Earlier presentation may reflect accelerated atherosclerosis but also implies lower frailty burden, fewer degenerative comorbidities, and greater physiological reserve. Despite the lipotoxicity and the deleterious effects of excess adipose tissue on the myocardium, patients with obesity appear to exhibit better preserved LVEF and less severe renal dysfunction, particularly in higher BMI categories [10,11].

Third, some studies suggest that obese patients may have larger coronary arteries, which can facilitate stent implantation and reduce the risk of restenosis or procedural complications [9]. Lower bleeding rates and shorter coronary care unit stays have also been reported after PCI [10,36]. This phenomenon could also be explained by the procoagulant and prothrombotic state associated with excess body weight, related to the increased adipose tissue and the subsequent activation of inflammatory and thrombotic pathways described above.

**Figure 2:**
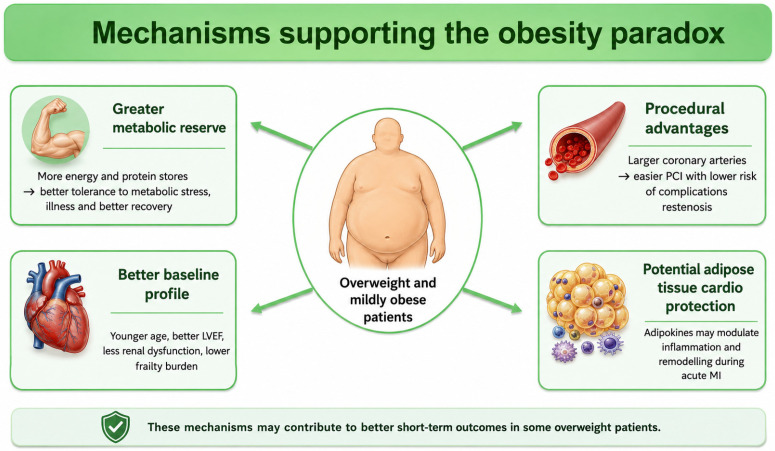
Mechanisms underlying the obesity paradox after ACS and PCI. Overweight and mildly obese patients may experience improved outcomes due to greater metabolic and nutritional reserves, younger age at presentation, lower frailty burden, and better preservation of cardiac and renal function. Additional factors, including larger coronary vessel size, lower procedural complication rates, and adipokine-mediated cardioprotective effects during acute MI, may also contribute. LVEF = Left ventricular ejection fraction, PCI = percutaneous coronary intervention, MI = myocardial infarction.

Finally, adipose tissue may exert transient cardioprotective effects during acute myocardial infarction (MI) through adipokine-mediated modulation of inflammation, apoptosis, and myocardial remodeling [12,20,37]. Indeed, adiponectin secreted by adipose tissue appears to play an important role in reducing infarct size, a phenomenon that has been extensively studied in murine models [38]. However, these mechanisms remain insufficient to explain the full magnitude of the observed clinical paradox. To further illustrate these findings, an additional summary figure is provided below (see Figure 2).

### 3.3. Why the Obesity Paradox May Be Misleading

The obesity paradox may largely reflect the limitations of BMI rather than a true protective effect of excess adiposity. BMI does not distinguish between fat mass and lean mass, nor does it provide information regarding visceral adiposity, sarcopenia, or nutritional status [14,39,40]. Several studies have shown that waist circumference, waist-to-hip ratio, and body fat percentage are more strongly associated with cardiovascular risk than BMI alone [39,40]. Furthermore, recent evidence suggests that body fat distribution is a stronger determinant of cardiovascular outcomes than overall body weight [3]. Patients with normal BMI but increased central adiposity, often referred to as “normal-weight central obesity,” have been shown to experience higher cardiovascular and all-cause mortality than individuals classified as overweight or obese by BMI alone [41,42]. Conversely, individuals with a higher BMI but lower visceral fat accumulation and preserved lean muscle mass may exhibit more favorable outcomes. These findings indicate that visceral adiposity, ectopic fat depots, and sarcopenia are key factors underlying cardiovascular risk and may partly explain the so-called obesity paradox. Therefore, the assessment of body composition and central obesity should complement BMI when evaluating prognosis in patients with cardiovascular disease. Advanced imaging techniques, including computed tomography (CT), magnetic resonance imaging (MRI), and dual-energy X-ray absorptiometry (DEXA), provide a more accurate characterization of adipose tissue distribution and body composition [43,44]. These approaches have demonstrated that visceral adiposity and reduced skeletal muscle mass are more closely associated with adverse cardiovascular outcomes than BMI itself, highlighting the importance of considering both fat distribution and muscle mass in cardiovascular risk stratification.

Sex and ethnicity further complicate the interpretation of BMI and may contribute to the apparent obesity paradox. For a given BMI, women generally have a higher percentage of total body fat but lower visceral adipose tissue than men, whereas men tend to accumulate more metabolically harmful visceral fat [45]. Consequently, BMI may underestimate cardiometabolic risk in women, for whom visceral adiposity appears to be more strongly associated with adverse cardiovascular outcomes than BMI itself [46]. Furthermore, a Chinese study of 664 patients with STEMI confirmed that mild obesity (BMI 30–35 kg/m^2^) had a paradoxical protective effect in men but not in women [47].

Similarly, substantial ethnic differences further highlight the limitations of BMI as a measure of cardiovascular risk. For a given BMI, variations in body composition and fat distribution exist across populations. Indeed, certain ethnic groups, particularly South Asians, tend to accumulate visceral fat and develop cardiometabolic complications at lower BMI levels [48,49]. In contrast, Black individuals often have greater lean mass and lower visceral adiposity for a given BMI [50]. Consequently, BMI may overestimate or underestimate cardiovascular risk depending on ethnicity, contributing to the heterogeneous associations observed between BMI and cardiovascular outcomes. These findings support the use of complementary measures of central adiposity and body composition when assessing cardiovascular risk.

Reverse causality also plays an important role. Patients with low BMI often include individuals with occult chronic disease, smoking-related weight loss, cachexia, or frailty, all of which independently worsen prognosis. Similarly, survival bias may select obese patients who survive long enough to undergo PCI, thereby enriching this group with healthier individuals [51].

**Figure 3:**
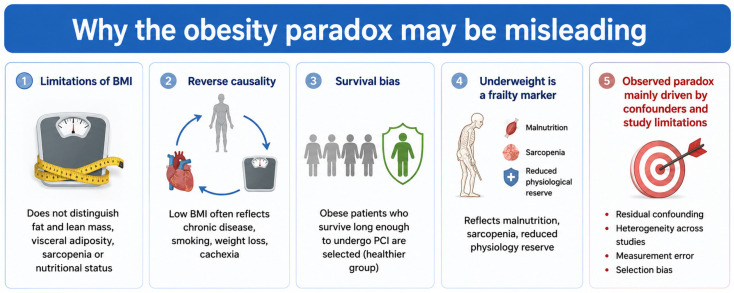
Limitations of BMI and the Role of Frailty, Malnutrition, and Reverse Causality. The apparent protective effect of obesity after ACS and PCI may largely reflect the limitations of BMI as a measure of adiposity, as it does not account for body composition, fat distribution, sarcopenia, or nutritional status. Reverse causality, survival bias, frailty, and chronic illness among low-BMI individuals may further contribute to the observed inverse association between BMI and clinical outcomes.

Underweight status consistently reflects frailty, malnutrition, sarcopenia, and reduced physiological reserve rather than simply low body weight [52,53]. This may explain why underweight patients systematically experience the highest mortality and MACE rates across studies. Rather than obesity being protective, poor outcomes in lean patients may drive much of the inverse association observed between BMI and prognosis. A complementary summary figure is presented below to highlight these observations (see Figure 3).

## 4. Clinical Evidence on MACE After PCI in ACS

### 4.1. MACE Definition Across the Available Literature

In the actual literature the definition of major adverse cardiovascular events (MACE) was not fully consistent, which represents an important source of heterogeneity when interpreting the obesity paradox. Most studies defined MACE using a composite endpoint including at least cardiovascular death and MI, while additional components such as stroke, early repeat revascularization, heart failure, unstable angina, or rehospitalization were included variably depending on the study. For instance, some authors considered only the classical “hard” cardiovascular endpoints (cardiovascular death, MI, and stroke), whereas others used broader definitions incorporating softer clinical outcomes such as angina recurrence or heart failure. This lack of uniformity makes direct comparison between studies more challenging and may partly contribute to the conflicting results reported in the literature [54].

According to contemporary cardiovascular literature, MACE is most commonly defined as a composite of cardiovascular death, MI, and stroke (the classical 3-point MACE), with some studies extending this to a 4-point MACE by adding urgent or unplanned revascularization [54]. Nevertheless, no universal consensus exists, and endpoint selection often depends on study design and clinical context [55].

The timing of MACE occurrence is an important factor when interpreting the obesity paradox. In ACS, early MACE usually refers to events occurring during hospitalization, at 30 days, or within the first 3 to 6 months, reflecting the direct short-term prognosis of the acute event. Chi et al. [56] showed that nearly 50% of recurrent MACE occurred within the first 90 days after ACS, while Steen et al. [57] reported that the risk of recurrent cardiovascular events was nearly six times higher immediately after discharge than beyond 1 year. In contrast, late MACE (>1 year) is more strongly influenced by the long-term progression of coronary artery disease, the effectiveness of secondary prevention strategies, and the evolution of comorbidities, rather than by the initial ACS event itself. This distinction is particularly relevant for the obesity paradox: Kadakia et al. [58] showed that obese patients had a lower risk of adverse events at 30 days, but this protective effect disappeared between 30 days and 1 year, suggesting that the obesity paradox may be mainly a short-term phenomenon rather than a true long-term protective effect.

### 4.2. Clinical Characteristics and Cardiovascular Risk Profile

Across our narrative research, patients with obesity consistently exhibited a higher burden of traditional cardiovascular risk factors. As described, numerous multicenter studies and meta-analyses have shown that obesity is strongly associated with several cardiovascular risk factors [17]. Indeed, hypertension, diabetes mellitus, and dyslipidemia are significantly more prevalent in obese individuals, as demonstrated in large registry-based cohorts such as the Israeli ACS registry [7] survey and Korean myocardial infarction registries [59]. Moreover, in a study made by Sharbati et al. [60], diabetes mellitus was more prevalent in patients with class I obesity, while hypertension and elevated LDL cholesterol levels predominated in class II obesity. In some cohorts, the prevalence of hypertension increased progressively across BMI categories, reaching nearly 42–52% in obese patients [61,62,63]. Similar patterns were observed in other cohorts such as the study of Hun-Tae Kim et al., where hypertension prevalence increased progressively across BMI categories (from 45.1% in underweight patients to 69.9% in obese patients; *p* < 0.001) [10].

Despite this unfavorable metabolic profile, obese patients were generally younger at the time of their ACS. Indeed, Chen Gurevitz et al. [14] showed that obese patients presented at a significantly younger age than underweight, normal-weight, and overweight patients (median age 61 vs. 70, 65, and 63 years, respectively; *p* < 0.001). Similarly, a retrospective analysis of 111,847 patients from the CRUSADE registry [35] demonstrated a strong inverse linear relationship between BMI and age at first NSTEMI (74.6 ± 14.3 years in underweight patients (BMI ≤ 18.5) compared with 58.7 ± 12.5 years in patients with severe obesity (BMI > 40)), representing a crude difference of nearly 12–16 years. This younger age at presentation likely reflects earlier development of atherosclerotic disease in obese populations, but it may also partly explain the comparable in-hospital prognosis observed despite their more adverse cardiovascular risk profile.

In addition to age differences, several studies reported better preserved cardiac and renal function in patients with higher BMI [11]. LVEF tended to increase with BMI, and renal impairment was more frequently observed in underweight or normal-weight individuals [10]. These findings suggest that obese patients may have greater physiological reserve at the time of the acute event.

Conversely, underweight patients consistently demonstrated features of frailty and higher clinical vulnerability. They were more likely to present with impaired renal function, reduced LVEF, and higher rates of comorbidity [10,11]. Importantly, studies incorporating nutritional indices, such as the prognostic nutritional index (PNI), revealed that a substantial proportion of patients were malnourished, including among those classified as overweight or obese [52]. This highlights a critical limitation of BMI as a marker of metabolic health and suggests that body composition and nutritional status may be more relevant determinants of prognosis [14,64].

To provide a clearer overview of these findings, a summary table of the current recent literature from retrospective and prospective study since 2020, describing the main clinical characteristics and cardiovascular risk profiles across BMI categories is presented below (Table 1).

### 4.3. Short-Term Outcomes After PCI

Short-term outcomes following PCI showed relatively consistent trends across the literature. Overweight and obese patients generally experienced lower rates of early MACE compared with normal-weight individuals, whereas underweight patients consistently had the highest event rates.

For example, in multicenter registry analyses, obese patients demonstrated lower rates of 30-day MACE in unadjusted analyses, while underweight patients exhibited the highest early risk [7]. Similarly, in smaller cohort studies, early MACE rates at 3 months were significantly reduced in overweight–obese patients compared with underweight–normal weight groups, with differences primarily driven by lower rates of reinfarction and repeat revascularization [11].

In most studies, in-hospital mortality remained low across all BMI categories and did not differ significantly, generally ranging from 0.6% to 2.3% [65]. These findings suggest that BMI-related differences in early outcomes are more strongly associated with morbidity rather than acute mortality. Nevertheless, differences in short-term clinical course were observed. In the study by Hun-Tae Kim et al., the length of stay in the coronary care unit decreased significantly with increasing BMI, from 5.3 days in underweight patients to 3.5 days in obese patients (*p* = 0.007) [10], supporting the notion of a more favorable early in-hospital course among obese individuals.

Additional findings included lower incidence of bleeding complications particularly among elderly obese patients [36]. These observations may be explained by differences in pharmacological management, including potential overdosing of antithrombotic therapies in low-weight individuals, as well as greater hemodynamic stability and metabolic reserve in patients with higher body mass. Another possible explanation for these findings is that patients with a higher BMI may present a more pronounced procoagulant and prothrombotic state, which could, to some extent, reduce the risk of hemorrhagic complications [21,22,23].

### 4.4. Long-Term Mortality and the Non-Linear Relationship with BMI

Long-term mortality outcomes were consistently reported across studies, with follow-up durations ranging from 12 to 60 months, and demonstrated a non-linear, U-shaped relationship between BMI and mortality. The lowest mortality rates were generally observed in overweight or mildly obese individuals, whereas underweight patients consistently exhibited the highest mortality risk across all cohorts.

In unadjusted analyses, class I obesity was often associated with reduced one-year mortality. This pattern was observed in the studies by Sharbati et al. and Chen Gurevitz et al. [7,60], where obese patients appeared to have better survival than normal-weight individuals. Similar findings were reported by Rahul Samanta et al., where long-term all-cause mortality occurred in 13.5% of normal-weight patients compared with 5.6% of overweight and 4.2% of obese patients (*p* < 0.01) [65].

However, this apparent protective effect of obesity was less consistent after multivariable adjustment. In several studies, obesity was no longer independently associated with improved survival, with adjusted hazard ratios approaching unity. For example, Chen Gurevitz et al. [7] reported that obesity was no longer significantly associated with lower one-year mortality after adjustment (adjusted HR 0.90; 95% CI 0.76–1.06; *p* = 0.20).

In contrast, overweight status more frequently remained associated with reduced mortality, even after adjustment. In the same study, overweight patients had a 24% reduction in one-year mortality (HR 0.76; 95% CI 0.66–0.88; *p* = 0.0002), while underweight patients had a significantly increased risk of death (HR 1.90; 95% CI 1.30–2.90; *p* = 0.002) [7]. Similarly, in a largest cohort of Chen Q-F et al., underweight patients had the highest mortality rate (7.2%), whereas overweight and obese patients showed significantly lower adjusted risks of death [52].

These findings suggest that the apparent obesity paradox may be driven less by a true protective effect of excess adiposity and more by confounding baseline characteristics such as younger age, earlier presentation, and differences in comorbidity burden. Overall, overweight status appears to confer the most consistent survival advantage, while underweight status remains a strong and reproducible predictor of poor long-term prognosis.

### 4.5. BMI and Major Adverse Cardiovascular Events

The association between BMI and MACE largely mirrored the patterns observed for mortality. Across the included studies, overweight and obese patients frequently showed lower crude rates of MACE, whereas underweight patients consistently experienced the highest event rates during both short- and long-term follow-up.

In unadjusted analyses, class I obesity was often associated with reduced MACE incidence. For example, in the study by Sharbati et al. [60], class I obesity was associated with lower rates of both MACE-3 and MACE-5 at one year. However, these associations were no longer significant after multivariable adjustment, suggesting that the apparent protective effect was largely influenced by baseline confounders rather than BMI itself.

Similarly, Firman et al. [11] reported that the two-year incidence of MACE was substantially lower in overweight–obese patients compared with underweight–normal-weight patients (24.1% vs. 39.9%; *p* < 0.001), mainly driven by lower rates of recurrent MI. However, these findings were not uniformly observed across all cohorts.

In the prospective study by Se-Jun Park et al., obesity (BMI ≥ 30 kg/m^2^) was not associated with a lower risk of MACE compared with normal-weight patients, regardless of diabetic status [59]. In contrast, moderate overweight (25 < BMI < 30 kg/m^2^) was associated with a significant reduction in MACE among non-diabetic patients (HR 0.78; 95% CI 0.62–0.97), while underweight diabetic patients had a significantly increased risk (HR 1.79; 95% CI 1.24–2.58) [59].

These findings suggest that the obesity paradox is far less consistent for MACE after multivariable adjustment and may largely reflect confounding rather than a true protective effect of excess adiposity. In the START-ANTIPLATELET Registry, Calabrò et al. [66] reported significantly lower rates of net adverse clinical events in overweight and obese ACS patients compared with normal-weight individuals (9.6% and 8.6% vs. 15.1%, *p* = 0.004) [66]. However, after adjustment for baseline characteristics, BMI was no longer an independent predictor of outcomes, indicating that the apparent benefit was mainly explained by differences in clinical profile and management rather than BMI itself. Similar findings were observed in large population-based studies, where the obesity paradox disappeared when pre-diagnostic weight rather than post-event BMI was considered, strongly suggesting reverse causality and residual confounding [51]. More recent evidence suggests that the obesity paradox may largely reflect the limitations of BMI rather than a true protective effect. Iliodromiti et al. [39] showed that while BMI displayed a J-shaped association with cardiovascular events, this relationship weakened after excluding patients with baseline comorbidities, whereas waist circumference, waist-to-hip ratio, and body fat percentage remained linearly associated with higher cardiovascular risk. Similarly, Coutinho et al. [40] found that central obesity was associated with increased mortality, while BMI was paradoxically inversely associated with mortality. These findings support the hypothesis that BMI is an imperfect marker of cardiovascular risk and that the apparent obesity paradox may largely reflect methodological bias, including lead-time bias, smoking, and differences in cardiorespiratory fitness.

In contrast, underweight status remains a strong and reproducible predictor of adverse cardiovascular outcomes. Underweight patients consistently exhibit the highest rates of mortality and MACE across both short- and long-term follow-up. In a prospective cohort of 57,574 post-MI patients followed for 17 years, Bucholz et al. [53] demonstrated significantly higher mortality in underweight individuals at all time points, from 30 days (25.2% vs. 16.4%) to 17 years (98.3% vs. 94.0%). Importantly, this excess risk remained significant even after adjustment for comorbidities, frailty markers, and nutritional biomarkers (adjusted HR 1.26 at long-term follow-up), and persisted even in patients without major comorbidities, suggesting that low BMI itself reflects a high-risk biological phenotype rather than simple cachexia. Similarly, Gurevitz et al. [7] reported the highest 1-year mortality among underweight ACS patients (24.8%), compared with 10.7% in normal-weight, 7.1% in overweight, and 7.5% in obese patients, with no improvement in outcomes over two decades in this subgroup. This unfavorable prognosis likely reflects frailty, sarcopenia, malnutrition, and reduced physiological reserve. Indeed, malnutrition and sarcopenia are now recognized as major determinants of poor cardiovascular prognosis, and low BMI may be better interpreted as a marker of biological vulnerability, advanced atherosclerotic burden, and reduced tolerance to ischemic stress rather than a benign phenotype.

### 4.6. Impact of Confounders and Effect Modifiers

A central finding across all studies in the available literature is the major influence of confounding variables on the relationship between BMI and cardiovascular outcomes. The apparent protective effect of obesity observed in unadjusted analyses was consistently attenuated after multivariable adjustment, suggesting that the obesity paradox may be largely explained by differences in baseline risk profile rather than by BMI itself. Age appears to be one of the most important determinants, as obese patients tend to present with ACS at a younger age and therefore have a more favorable prognosis at baseline. Adjustment for age, LVEF, renal function, and cardiovascular risk burden substantially reduced the strength of this association [11,65].

In several cohorts, overweight status—but not obesity—remained independently associated with improved outcomes after adjustment, whereas underweight status consistently conferred the highest risk of mortality and MACE. Subgroup analyses further highlighted important effect modifiers. In younger patients, overweight and obesity were associated with lower rates of adverse outcomes (adjusted OR for obesity 0.51; 95% CI 0.34–0.75), whereas in elderly populations, the obesity paradox was attenuated or disappeared, and in some studies obesity was even associated with worse outcomes [36]. This suggests that the prognostic impact of BMI varies significantly across age groups.

Nutritional status also emerged as a major determinant of prognosis. Malnutrition was independently associated with a marked increase in mortality risk (HR 2.64; 95% CI 2.24–3.12), and the obesity paradox appeared to be most evident among malnourished patients, while it was absent in nutritionally preserved individuals. [52] This supports the hypothesis that the excess risk observed in lean patients may reflect frailty, sarcopenia, and catabolic states rather than low BMI itself [53]. Across all subgroups, underweight status remained consistently associated with the poorest prognosis, reinforcing its importance as a high-risk clinical phenotype.

## 5. Discussion

This narrative review provides a comprehensive synthesis of the relationship between BMI and clinical outcomes in patients undergoing PCI for ACS. Across the nine included studies, a complex and non-linear association was consistently observed, characterized by an apparent obesity paradox in which overweight and mildly obese patients appeared to experience better short- and long-term outcomes than normal-weight or underweight individuals.

However, this paradox must be interpreted cautiously. Although obesity is a well-established risk factor for the development of coronary artery disease and ACS, its prognostic impact after PCI appears to be strongly influenced by confounding factors. In most studies, the apparent protective effect of obesity was markedly attenuated or disappeared after multivariable adjustment for age, LVEF, renal function, cardiovascular risk burden, and nutritional status [11,36,59,60,65]. These findings strongly suggest that obesity itself does not confer an independent survival advantage, but rather reflects differences in baseline clinical profile.

Obese patients consistently presented with a less favorable metabolic profile, including higher rates of hypertension, diabetes mellitus, and dyslipidemia [66,67,68]. Despite this, they were generally younger at the time of ACS presentation and often had better preserved cardiac and renal function compared with leaner patients [66]. Younger age at presentation appears to be one of the main explanations for the lower crude mortality observed in overweight and obese patients. This lead-time effect may partially explain the obesity paradox, as obese individuals develop coronary disease earlier and therefore receive earlier diagnosis and treatment before the onset of advanced frailty or organ dysfunction [3,35].

In addition to the limitations of BMI, several methodological biases may contribute to the observed obesity paradox, including reverse causality, survival bias, and collider bias. Patients with low BMI may include individuals with occult chronic disease, cachexia, or smoking-related weight loss, all of which are independently associated with worse cardiovascular outcomes. Conversely, patients with obesity who survive long enough to develop coronary artery disease and undergo PCI may represent a selected subgroup with greater physiological reserve and a more favorable risk profile. Furthermore, restricting analyses to patients who have already experienced a cardiovascular event or undergone PCI may introduce collider bias, as BMI becomes associated with other prognostic factors within this selected population, potentially creating a spurious protective association. Supporting this interpretation, Mendelian randomization studies have generally failed to demonstrate a causal survival benefit of higher adiposity, suggesting that at least part of the obesity paradox may reflect residual confounding, selection bias, and the limitations of BMI as a measure of cardiovascular risk [10].

In contrast, underweight status consistently emerged as the strongest predictor of poor prognosis. Across all studies, underweight patients had the highest rates of mortality and MACE, both during hospitalization and at long-term follow-up. This finding likely reflects the combined effects of frailty, sarcopenia, malnutrition, chronic inflammation, and reduced physiological reserve rather than low BMI alone [52,68]. Importantly, malnutrition was frequently present even among overweight and obese patients, highlighting that BMI as a standalone metric is insufficient to assess nutritional risk. Several studies showed that the obesity paradox was predominantly observed in malnourished patients, suggesting that the excess mortality of lean individuals may drive much of the inverse association between BMI and outcomes [27].

Short-term outcomes after PCI further support this interpretation. In-hospital mortality remained generally low and did not significantly differ across BMI categories, whereas differences were mainly observed in morbidity-related outcomes such as MACE, bleeding complications, and coronary care unit length of stay [7,10,11]. Obese patients often had fewer bleeding events and shorter ICU stays, which may partly reflect better hemodynamic tolerance, greater metabolic reserve, and reduced relative overdosing of antithrombotic therapies compared with leaner patients.

Long-term follow-up demonstrated a consistent U-shaped relationship between BMI and mortality, with the lowest risk typically observed in overweight or mildly obese patients and the highest risk in underweight individuals. Class I obesity was often associated with lower crude mortality, but this effect frequently disappeared after adjustment, whereas overweight status more often remained independently associated with improved survival [12,19,26,27,30]. These findings suggest that the obesity paradox reflects age, metabolic reserve, clinical management, and selection bias more than a true protective effect of excess adiposity.

These complex biological interactions further highlight the limitations of relying solely on BMI to characterize adiposity and cardiovascular risk. The limitations of BMI as an anthropometric marker must also be emphasized. BMI does not differentiate between fat mass and lean mass and provides no information regarding visceral adiposity or fat distribution [14]. Several contemporary studies have shown that the obesity paradox is largely dependent on the parameter used to assess adiposity. While BMI often demonstrates a U- or J-shaped relationship with outcomes, measures such as waist circumference, waist-to-hip ratio, and body fat percentage show a more linear positive association with cardiovascular risk, suggesting that BMI may underestimate true cardiovascular risk [14,68]. Similarly, normal-weight individuals with increased abdominal adiposity have consistently been shown to experience worse cardiovascular outcomes than overweight or obese individuals without central obesity.

Beyond anthropometric limitations, heterogeneity in metabolic health among individuals with similar BMI values further complicates risk stratification. The concept of metabolically healthy obesity (MHO) further highlights the limitations of BMI-based risk assessment [69]. MHO describes individuals with obesity who exhibit few or no metabolic abnormalities despite excess adiposity [69]. However, the definition of MHO remains heterogeneous across studies, and accumulating evidence suggests that this phenotype is not entirely benign. Long-term cohort studies have demonstrated that individuals with MHO remain at increased risk of cardiovascular disease, heart failure, and all-cause mortality compared with metabolically healthy normal-weight individuals, particularly with prolonged follow-up [70,71]. Nevertheless, cardiovascular risk remains lower in individuals with MHO than in those with metabolically unhealthy obesity [72]. These findings suggest that the apparent protection observed in some obese populations may reflect differences in metabolic health and adipose tissue distribution rather than excess body weight itself.

Advanced imaging modalities, including computed tomography, magnetic resonance imaging, and dual-energy X-ray absorptiometry, further demonstrate that visceral adipose tissue, epicardial adipose tissue, and reduced skeletal muscle mass are more closely associated with adverse cardiovascular outcomes than BMI itself [43,44]. Moreover, the interpretation of BMI varies according to sex and ethnicity, as important differences in body composition and fat distribution exist across populations. For a given BMI, women generally have a higher body fat percentage than men, whereas South Asian individuals tend to accumulate visceral fat and develop cardiometabolic complications at lower BMI levels, while Black individuals often have greater lean mass and lower visceral adiposity [49,50,73]. Consequently, BMI may substantially overestimate or underestimate cardiovascular risk depending on the population studied.

Although epidemiological findings suggest that the obesity paradox is largely explained by confounding and methodological biases, several biological mechanisms have nevertheless been proposed to account for the observed associations. From a pathophysiological perspective, adipose tissue should not be considered merely as an energy storage compartment but rather as an active endocrine organ [19]. Through the secretion of adipokines such as adiponectin, leptin, resistin, and inflammatory cytokines, adipose tissue may exert complex effects on systemic inflammation, endothelial function, thrombosis, insulin sensitivity, and myocardial remodeling [20]. Not all adipose depots exert identical biological effects. In particular, EAT, which lies in direct contact with the myocardium and coronary arteries, may exert both protective and detrimental cardiovascular effects depending on its metabolic and inflammatory profile [15,29]. This duality further illustrates the complexity of adipose tissue biology and suggests that adipose tissue distribution may be more relevant than BMI-based classification when assessing cardiovascular risk. In the acute setting of ACS, some of these mechanisms may transiently contribute to improved tolerance to ischemic stress or vascular injury, potentially participating in the so-called obesity paradox [37]. However, these potential short-term protective effects remain insufficient to fully explain the observed clinical patterns and are likely outweighed by the well-established long-term adverse consequences of excess adiposity, including accelerated atherosclerosis, metabolic dysfunction, and chronic cardiovascular risk [18].

Differences in therapeutic management may also contribute to these observations. Overweight and obese patients may be more likely to receive guideline-directed medical therapy, including beta-blockers, statins, and dual antiplatelet therapy, and may demonstrate better adherence during follow-up [7,10,36]. In contrast, underweight or frail patients may be undertreated because of concerns regarding bleeding risk, hypotension, or intolerance to aggressive therapy, which may further worsen their prognosis.

Recent therapeutic advances also challenge the concept of a protective effect of obesity. In the SELECT trial [74], semaglutide significantly reduced major adverse cardiovascular events, cardiovascular mortality, and all-cause mortality in overweight and obese patients with established cardiovascular disease, providing compelling evidence that intentional reduction in excess adiposity improves cardiovascular outcomes [74,75]. Notably, these benefits appeared to extend beyond weight loss alone, suggesting additional cardiometabolic, anti-inflammatory, and pleiotropic effects of GLP-1 receptor agonists.

Similarly, ongoing outcome trials evaluating newer incretin-based therapies, such as tirzepatide, are expected to further clarify the relationship between adiposity reduction and cardiovascular risk [75,76,77]. In parallel, SGLT2 inhibitors have demonstrated substantial cardiovascular and renal benefits across a broad spectrum of cardiometabolic diseases, supporting a treatment paradigm focused on improving cardiometabolic health rather than viewing excess body weight as potentially protective [78,79]. Collectively, these findings challenge the notion that obesity is inherently protective and further reinforce the importance of targeting excess adiposity and its metabolic consequences.

From a clinical perspective, these findings should not be interpreted as evidence that obesity is protective. Instead, they support a more nuanced approach to cardiovascular risk stratification beyond BMI alone [3,80]. Particular attention should be given to underweight and malnourished patients, who represent a particularly vulnerable subgroup and may benefit from targeted nutritional assessment and individualized therapeutic strategies [42]. Tools such as the Controlling Nutritional Status (CONUT) score may improve prognostic evaluation and help identify patients at high risk of MACE despite apparently preserved BMI [81,82]. Other studies confirm that the risk of MACE increases proportionally to the severity of malnutrition, while recognizing that even a patient with a high BMI can have a malnourished status [82,83,84,85,86].

Future research should focus on integrating more precise markers of body composition, frailty, sarcopenia, and nutritional status into prognostic models for ACS patients undergoing PCI. Rather than representing a true protective effect of excess adiposity, the obesity paradox in ACS likely reflects the detrimental prognostic impact of frailty, malnutrition, and reduced physiological reserve among leaner patients, highlighting the need to move beyond BMI-based risk stratification in contemporary cardiovascular practice.

## 6. Limitations

Certain limitations should be acknowledged when interpreting the findings of this narrative review. First, as a narrative review, it is inherently subject to selection and interpretation bias and does not provide the quantitative synthesis of a systematic review or meta-analysis. Second, the available literature on the obesity paradox remains highly heterogeneous with regard to patient populations, definitions of obesity, study designs, and clinical endpoints. In addition, many studies rely on BMI as the primary measure of adiposity, despite its well-recognized inability to distinguish between fat mass and lean mass or to account for fat distribution, visceral adiposity, sarcopenia, and ethnic- or sex-related differences in body composition. Consequently, the observed associations between BMI and cardiovascular outcomes should be interpreted with caution. Emerging approaches, including advanced body composition imaging, epicardial adipose tissue assessment, and novel echocardiographic parameters such as myocardial work indices, may provide a more accurate characterization of cardiovascular risk, but their role in routine clinical practice and risk stratification requires further validation. Finally, the rapid development of cardiometabolic therapies, including GLP-1 receptor agonists and SGLT2 inhibitors, may further refine our understanding of the obesity paradox in the coming years.

## 7. Conclusions

This narrative review confirms that the relationship between BMI and cardiovascular outcomes after ACS treated with PCI is complex and non-linear. Although overweight and, in some cases, obesity is associated with lower crude mortality and MACE rates, these associations are largely attenuated after multivariable adjustment. In contrast, underweight status consistently emerges as a strong and independent marker of poor prognosis. These findings suggest that the so-called obesity paradox likely reflects residual confounding and the impact of age, cardiac function, and nutritional status rather than a true protective effect of excess body weight. Systematic nutritional assessment should therefore complement BMI in risk stratification following acute coronary syndromes.

## Figures and Tables

**Table 1:** Summary table with overview of the actual literature.

Authors	Location of the Study	Study Design	Age (Year)	Male	Mean FUP (Year)	Full Sample Size	Number of Obese Patients	Obesity Paradox Demonstrated	Type of ACS	Effect Estimate
**M. Sharbati et al.**	Iran	Retrospective observational study, single center	57 ± 11.5	78%	1	2397	382	No	STEMI	OR 1.49 (class II obesity, mortality)
**Se-Jun Park et al.**	Korea	Prospective cohort study, national cohort	60.8 ± 9.7/ 64.4 ± 9.5 *	63%	5.4 ± 3.7	6978	250	Partial ^1^	All ACS	HR 0.78; NS in diabetic
**Chen Gurevitz et al.**	Israeli	Retrospective observational study, multicentric	Median 61–71	78%	1	13,816	3567	Partial ^2^	All ACS	HR 0.76 (overweight, significant); NS in obese (HR 0.90)
**D. Firman et al.**	Indonesia	Retrospective observational study, single center	57 ± 10.55/ 57.2 ± 10.7 ^#^	86%	2.2	400	212	Yes	STEMI with PCI	OR 2.32 (higher risk in low BMI)
**Hun-Tae Kim et al.**	Korea	Prospective observational study, multicentric	81	55%	1	2489	148	Yes	Not mentioned	HR 0.20 (95% CI 0.06–0.69)
**Rahul Samanta et al.**	Australia	Prospective observational study, single center	56.67 ± 11.52	83%	2.2	2913	132	Yes	STEMI	HR 2.7 (normal vs. obese)
**Alexandra-Cătălina Frisan et al.**	Romania	Prospective observational study, single center	59 ± 11	77.7%	1.1	143	53	No	STEMI	NS (MACE 15.1% vs. 24.4%, *p* = 0.185)
**Jinwen Wang et al.**	China	Prospective observational study, single center	58 ± 11.8	83.1%	4.9	1429	417	Partial ^3^	STEMI with PCI	NS in elderly/OR 0.508 (obese vs. normal)
**Qin-Fen Chen et al.**	China	**Prospective observational study, single center**	64.8 ± 11.3	69.2%	2.4	21,651	983	Partial ^4^	ACS with PCI	HR 0.60 (obese vs. normal, adjusted)/NS in nourished

^1^: varies by diabetes statues; ^2^: varies by stage of obesity; ^3^: varies by age; ^4^: varies by nutrition statues; * non-diabetic/diabetic; ^#^ overweight-obese/underweight-normal.

## Data Availability

No new data were generated in this manuscript.

## Abbreviations

The following abbreviations are used in this manuscript:

PCI	Percutaneous coronary intervention
ACS	Acute coronary syndrome
MACE	Major adverse cardiovascular events
BMI	Body mass index
LVEF	Left ventricular ejection function
WHO	World Health Organization
MI	Myocardial infarction
EAT	Epicardial adipose tissue
ATP	Adenosine triphosphate
CABG	Coronary artery bypass grafting
MHO	metabolically healthy obesity
